# PILOT FEASIBILITY STUDY OF EMR-BASED IDENTIFICATION OF PSYCHOLOGICAL DISTRESS AROUND RECENT DEMENTIA DIAGNOSES

**DOI:** 10.1093/geroni/igae098.1381

**Published:** 2024-12-31

**Authors:** Sarah Bannon, Ayushi Divecha, Kristen Dams-O’Connor, Alex Federman

**Affiliations:** Icahn School of Medicine at Mount Sinai, New York, New York, United States; Icahn School of Medicine at Mount Sinai, New York, New York, United States; Icahn School of Medicine at Mount Sinai, New York, New York, United States; Icahn School of Medicine at Mount Sinai, New York, New York, United States

## Abstract

ADRDs have a significant impact on individuals, families, and systems. Though technological advances have enabled earlier diagnoses, early psychological distress experienced around the time diagnosis is largely overlooked. Psychological distress is an important early intervention target given its pronounced negative impact on ADRD adjustment. Interventions are limited by our insufficient understanding of psychological distress experienced around the time of ADRD diagnoses. Our primary objective was to investigate the feasibility of identifying EMR-based psychological distress in a diverse sample of individuals with recent ADRD diagnoses. We conducted secondary data analysis of EMR records obtained from the Mount Sinai Data Warehouse (MSDW) and identified N=12249 individuals with recent ADRD diagnoses after age 65. ICD-10 diagnoses of depression and anxiety were identified in (32.73%) and (27.58%) of the sample, respectively. We also examined scores above the standard cut-offs for clinically elevated distress using self-report measures of depression [PHQ-9 (>=10)] and anxiety [GAD-7 (>=10)]. Unfortunately, these measures were assessed in a very small proportion of the sample at the visit of dementia diagnosis. Out of 509 (4.1%) individuals with self-report measures available, 113 (22%) indicated clinically elevated distress.. Structured data elements can be used to identify distress in the context of dementia diagnosis, though they are limited in their administration and may only offer the potential to identify distress in a subset of individuals. Our findings emphasize the importance of further exploration of unstructured data elements to identify distress.

